# Live Imaging Provides New Insights on Dynamic F-Actin Filopodia and Differential Endocytosis during Myoblast Fusion in *Drosophila*

**DOI:** 10.1371/journal.pone.0114126

**Published:** 2014-12-04

**Authors:** Shruti Haralalka, Claude Shelton, Heather N. Cartwright, Fengli Guo, Rhonda Trimble, Ram P. Kumar, Susan M. Abmayr

**Affiliations:** 1 Stowers Institute for Medical Research, Kansas City, Missouri, 64110, United States of America; 2 Department of Anatomy and Cell Biology, University of Kansas School of Medicine, Kansas City, Kansas, 66160, United States of America; University of Valencia, Spain

## Abstract

The process of myogenesis includes the recognition, adhesion, and fusion of committed myoblasts into multinucleate syncytia. In the larval body wall muscles of *Drosophila*, this elaborate process is initiated by Founder Cells and Fusion-Competent Myoblasts (FCMs), and cell adhesion molecules Kin-of-IrreC (Kirre) and Sticks-and-stones (Sns) on their respective surfaces. The FCMs appear to provide the driving force for fusion, via the assembly of protrusions associated with branched F-actin and the WASp, SCAR and Arp2/3 pathways. In the present study, we utilize the dorsal pharyngeal musculature that forms in the *Drosophila* embryo as a model to explore myoblast fusion and visualize the fusion process in live embryos. These muscles rely on the same cell types and genes as the body wall muscles, but are amenable to live imaging since they do not undergo extensive morphogenetic movement during formation. Time-lapse imaging with F-actin and membrane markers revealed dynamic FCM-associated actin-enriched protrusions that rapidly extend and retract into the myotube from different sites within the actin focus. Ultrastructural analysis of this actin-enriched area showed that they have two morphologically distinct structures: wider invasions and/or narrow filopodia that contain long linear filaments. Consistent with this, formin Diaphanous (Dia) and branched actin nucleator, Arp3, are found decorating the filopodia or enriched at the actin focus, respectively, indicating that linear actin is present along with branched actin at sites of fusion in the FCM. Gain-of-function Dia and loss-of-function Arp3 both lead to fusion defects, a decrease of F-actin foci and prominent filopodia from the FCMs. We also observed differential endocytosis of cell surface components at sites of fusion, with actin reorganizing factors, WASp and SCAR, and Kirre remaining on the myotube surface and Sns preferentially taken up with other membrane proteins into early endosomes and lysosomes in the myotube.

## Introduction

Myoblast fusion is initiated by cell recognition and adhesion, and culminates in loss of membrane between the adjoining cells and absorption of the nucleus into the developing syncytia. In the body wall muscles of the *Drosophila* embryo, fusion occurs asymmetrically between Founder Cells (FC) and Fusion-Competent Myoblasts (FCMs) or, subsequently, between developing myotubes and FCMs. Numerous studies have revealed F-actin foci at the contact site between FCMs and either FCs or myotubes [1], [2] that disappear just prior to fusion [2]. These actin foci form in the center of a ring of Sticks-and-stones (Sns) and Kin-of-IrreC (Kirre), cell surface molecules that are essential for myoblast fusion [1], [3]. More recent studies have clarified that the actin foci are distributed asymmetrically at sites of fusion, localizing preferentially to the FCM [3], [4], [5] and are coincident with finger-like structures that protrude from the FCM into the myotube [3], [4], [5].

The asymmetry at the FC:FCM and myotube:FCM site of fusion is initially established at the time of myoblast recognition and adhesion by members of the Immunoglobulin Superfamily (IgSF). Sns is expressed exclusively on the surface of the FCMs, while Kirre and Roughest (Rst/IrreC) play redundant roles on the surface of the FC [6], [7], [8]. Hibris is a paralog of Sns and also restricted to the FCMs. While it is not essential for somatic muscle development [9], [10] studies have shown that it is capable to directing limited fusion in the absence of Sns [11]. The relative affinities of these cell surface proteins for each other [12] is one mechanism by which the directionality of the fusion process is ensured. Studies have also shown that Antisocial (Ants)/Rolling pebbles (Rols) stabilizes Kirre at the myotube surface through interaction with its cytoplasmic domain [13]. Subsequent to their heterotypic interaction at the cell surface, Kirre and Sns function as signaling molecules to mediate intracellular events [14] that lead to actin polymerization and fusion.

The nucleation promoting complexes Vrp1/WASp and WAVE/SCAR, which regulate F-actin branching by the Arp2/3 complex, mediate formation of the actin focus and are required for myoblast fusion [2], [15], [16], [17], [18], [19], [20]. SCAR, a subunit of the WAVE/SCAR complex, appears to be required in both cells types since expression in neither the FCs nor the FCMs fully rescues the muscle defects of *SCAR* mutant embryos [5]. In contrast, the Vrp1/WASp complex appears to function exclusively in the FCMs, which preferentially express Vrp1/WIP/Sltr [17], [18]. This complex is recruited to sites of contact via interaction with the SH2/SH3 adaptor protein Dreadlocks (Dock), with interacts with the cytoplasmic domain of Sns [21]. Some functional redundancy between these NPFs has been suggested by the observation that *Vrp1*, *SCAR* double mutants are more severe than single mutants [15] and that loss of both *Vrp1* and *SCAR* results in the absence of actin foci [5]. The observation that foci are still present in embryos doubly mutant for *kette* and *WASp*, or *Vrp1* and dominant negative SCAR [16] has also raised the question of whether other pathways, such as the formin-dependent polymerization of bundled actin, may also contribute to actin foci.

To gain new insights into the process of myoblast fusion, we have imaged the formation of the dorsal pharyngeal musculature (DPM) in living *Drosophila* embryos. We have first established that each of these muscles forms through fusion of a single FC with multiple FCMs, and that fusion relies on the same constellation of genes as the well-studied body wall muscles. As anticipated, cell surface IgSF proteins are present at points of cell contact, and nuclei become positioned near the membrane of the resulting syncytia. We now find from time-lapse imaging with F-actin and membrane markers that invasive structures protruding into the myotube are highly dynamic prior to the actual fusion event and precedes the internalization of membrane proteins at sites of fusion that colocalize, in fixed tissue, with Sns. Through differential endocytosis, Kirre and actin nucleation markers, SCAR and WASp, remain on the myotube surface while Sns is preferentially taken up into early endosomes and lysosomes. We further show, using TEM and live imaging the presence of two types of invasive structures that project from the FCM into the myotubes; wide structures at the base of the invasion that are coincident with dense actin foci, and thinner filopodia-like protrusions that rapidly extend and retract from the foci. Moreover, EM of high-pressure freeze-freeze substitution (HPF-FS) samples has revealed the presence of bundled linear filaments in these protrusions. The hypothesis that the dynamic membrane and actin protrusions reflect the rapid polymerization and depolymerization of linear actin filaments is also consistent with the presence of formin Diaphanous (Dia) in these filopodia-like protrusions. Therefore, rather than rename these structures as distinct from conventional filopodia, we follow the suggestion of Yang and Svitkina [22] and refer to these simply as filopodia. We also find branched actin nucleator, Arp3, to be present at sites of actin enriched in the FCM. Our gain-of-function studies with Dia and the loss-of-function studies on Arp3 both show a decrease in F-actin foci and prominent filopodia, suggesting that a compromise of the branched actin pathway leads to an increased formation of linear actin. Together, these data are consistent with a model in which Dia-based filopodia and Arp2/3 based branched actin structures coexist at sites of fusion, possibly to scan for membrane subdomains or generate sites of high negative membrane curvature prior to generating the force for invasion and fusion.

## Materials and Methods

### Fly stocks & genetics

Fly stocks included *UAS-Rab5.S43N-YFP, UAS-Rab5-YFP, UAS-Rab5.Q88L-YFP, UAS-Rab4-YFP, UAS-Rab11-YFP, UAS-Actin-GFP* (Bloomington Stock Center), *UAS-LAMP-GFP* [23], *rP298-lacZ* [24], *sns-nls-mCherry* [3], *UAS-Actin-mCherry* [25], *Arp3* *^schwächling^* [15], *kette^J4–48^* [26], *wip^D30^* [18], *Df(1)w [67]k [30]* [7], *sns^XB3^* [6], *mbc^D11.2^* [27], *UAS-Dia* and *UAS-Dia*-Δ *DAD* [28], *mef2-Gal4* [29], *sns-Gal4* [30], *twi-Gal4* and *UAS-gap-GFP* [3] have been described. The UAS-gap-GFP plasmid was modified to generate UAS-gap-mCherry. The enhancer described for *twi-Gal4[108.4]* [31] was used to generate additional *twi-Gal4* transgenes, which were recombined in multiple copies for live imaging. EGFP-LifeAct-attB construct was generated by digesting out EGFP-LifeAct from the plasmid, pEGFP-N1_LifeAct, and inserting it into the pUAST-attB vector.

### Immunohistochemistry and Immunofluorescence

Primary antisera are as noted: rabbit anti-Sns-Cyto (1∶200) [12], guinea pig anti-N-Kirre (1∶250) [32], rabbit anti-dMef2 (1∶200) [33], chicken anti-β-galactosidase (1∶200, Abcam), rabbit anti-Dia (1∶250) [34], rabbit anti-Arp3 (1∶200) [35], guinea pig anti-SCAR (1∶200) [36], guinea pig anti-WASp (1∶200) [36], rabbit anti-GFP (1∶250, AbCam), and rabbit anti-DsRed (1∶200, Clontech). Mouse monoclonal antibodies included anti-Discs-large (Dlg) (1∶10, clone 4F3) from the Developmental Studies Hybridoma Bank and anti-myosin heavy chain (MHC, 1∶1000, gift of D. Kiehart). Colorimetric detection, fluorescent detection and visualization of F-actin with phalloidin have been described [3].

### Confocal microscopy

Confocal microscopy on fixed embryos was performed using a Zeiss LSM 510 Meta microscope, maintaining a pinhole of 1.0 airy units. Images were processed post collection where necessary with ImageJ using the rolling ball method at a 30–50 pixel radius for background subtraction, and Gaussian blur at a 0.5–1.00 pixel radius. Data collection for quantitative image analysis used a Zeiss LSM 510-Meta or a Zeiss LSM 780 using a Plan-Apochromat 63x/1.40 Oil DIC infinity corrected objective and a 0.28 µm step distance between Z sections. Collection settings were held constant for all images associated with a given experiment.

For live imaging, stage 13 embryos were processed as described [37]. Actin-mCherry and gap-GFP were excited simultaneously with 488 and 561 nm laser lines, and the emission collected through BP 575–615 IR and BP 505–550 filters respectively using a 40X/1.2NA C-Apochromat water objective. The pixel dwell time was 1.61 µs, averaging of four, and Z step of 1 µm. Actin-GFP and gap-mCherry were also excited simultaneously with 488 and 561 nm laser lines, and emission collected through LP 580 filters and BP 505–540 IR respectively. The pixel dwell time was 6.39 µs, averaging of 1 and Z step of 1 µm. The Avalanche photodiode (APD) imaging module of a Zeiss Confocor 3 was used in single photon counting mode. The APD time series were then converted to 32 bit images and processed using a Gaussian blur of 1.0 radius in ImageJ (v. 1.37; National Institutes of Health). GFP-LifeAct-attP40 and gap-mCherry live imaging was performed using the Zeiss LSM 780 microscope. The reporters were excited sequentially with 488 and 561 nm laser lines and emission collected through filters 499–560 and 568–630 respectively with an averaging of four and Z step of 1 µm. The time series data was processed similar to the APD time series.

Spinning-disk confocal microscopy (UltraVIEW; PerkinElmer) included an inverted microscope (Axiovert 200 M; Carl Zeiss) attached to a spinning-disk confocal system (CSU-X1; Yokogawa Corporation of America). The data was collected with a Plan-Apochromat 63x/1.20 water objective using a Hamamatsu EMCCD (C9100-23B) camera. Z-series were acquired every 12 seconds, with Z steps of 0.5 µm and imaging field resolution at 512×512 pixels. Actin-GFP and gap-mCherry were excited sequentially with 488 and 561 nm laser lines, and emission collected through BP 525–550 and BP 615–670 filters respectively. Images were processed using ImageJ. Final images were binned, background subtracted and contrast adjusted for clarity and its movie was generated in ImageJ at 5 frames per seconds.

For confocal microscopy prior to conventional TEM, embryos were fixed at room temperature in 1∶1 mix of heptane and fixative containing 2% formaldehyde, 2.5% glutaraldehyde in 0.1M sodium cacodylate buffer for 30 minutes. Stage 14 embryos were then hand devitellinized and incubated overnight at 4°C in fixative plus 1∶10 phalloidin Alexa-647. Each embryo was then immobilized on a 22×60 mm, # 1.5 coverslip with 1% low melting agarose and the DPM was imaged confocally using a 40X/1.2NA C-Apochromat water objective and Z step of 0.5 µm. Only DPMs having myoblasts with filopodial protrusions were further processed for visualization of the region containing the actin focus by conventional TEM.

All images were rescaled in Photoshop to publication size and zoomed by nearest neighbor or bilinear interpolation for easy viewing. Three dimensional movies of the confocal data (Movie S1–S5, S8, S9) were generated by first isosurfacing myotubes manually in Imaris using the tropomyosin signal as a guide. Animations were made using a frame rate of 5 frames per second and saved as an uncompressed avi file. The avi files were converted to.mov files using Handbrake.

### Electron microscopy

For conventional transmission electron microscopy (TEM), previously fixed embryos were post fixed in 1% osmium tetroxide in 0.1M sodium cacodylate, embedded in Epon resin, and ultrathin 50–70 nm sections examined (80 kV; Tecnai Spirit; FEI).

For HPF-FS electron microscopy, stage 14 embryos were frozen on a Leica EM-Pact at 2100 bar, and transferred under liquid nitrogen into 2% osmium tetroxide/0.1% uranyl acetate/acetone to an automated freeze-substitution apparatus (AFS) (Leica, Vancouver). Samples were embedded in an acetone/Epon mixture and transferred to 100% Epon as described [38]. Sectioning and staining were done as described for conventional EM, and serial section images aligned using AutoAligner (Bitplane).

### Quantitation of nuclei and myotube number and calculation of Fusion index

To monitor the number of nuclei per myotube and number of myotubes per individual in the DPM, nuclei of *wild-type*, late stage 16 embryos were counted manually from confocal Z-series images of embryos immunostained with dMef2 to monitor nuclei and Dlg to outline the membrane. To determine whether a single FC was present for each myotube in the DPM, and reflected the number of myotubes, the total number of FC was quantitated in fusion-defective *mbc* mutant embryos expressing the FC marker rp298lacZ. Immunostaining for β-gal and Dlg marked the nuclei and membrane respectively.

Fusion index was calculated according to guidelines utilized for mammalian myoblasts in culture, as the percentage of nuclei in multinucleate syncytia (2 or more nuclei) vs. the total number of nuclei in myoblasts and syncytia [3], [39], [40]. For this measurement, the Spots function in Imaris (Bitplane) was used in automatic mode to calculate the total number of nuclei in stage 15 embryos using the fluorescent signal of dMef2 as a guide. Calculation of nuclei within the myotubes was carried out manually, using tropomyosin to monitor the myotube plasma membrane.

### Quantitative of actin volume and frequency of filopodia

The region containing the actin focus was manually selected using the Crop 3D function in Imaris, excluding individual filopodia that extended outside of the box. The volume was then determined from voxel intensity using the automatic mode of the Isosurface function as described [3].

The number of filopodia was assessed manually for each actin-enriched focus. A filopodia was interpreted as any pointed projection that extends into the myotube from the base of the actin-enriched edge of the FCM protruding. To eliminate bias, quantitation of actin volume and filopodial frequency comparing *Arp3* mutants and *wild-type* samples was done double blind in coded samples.

## Results

### The DPM comprises a tightly associated array of myotubes, each generated by fusion of a single FC with multiple FCMs

**Figure 1:**
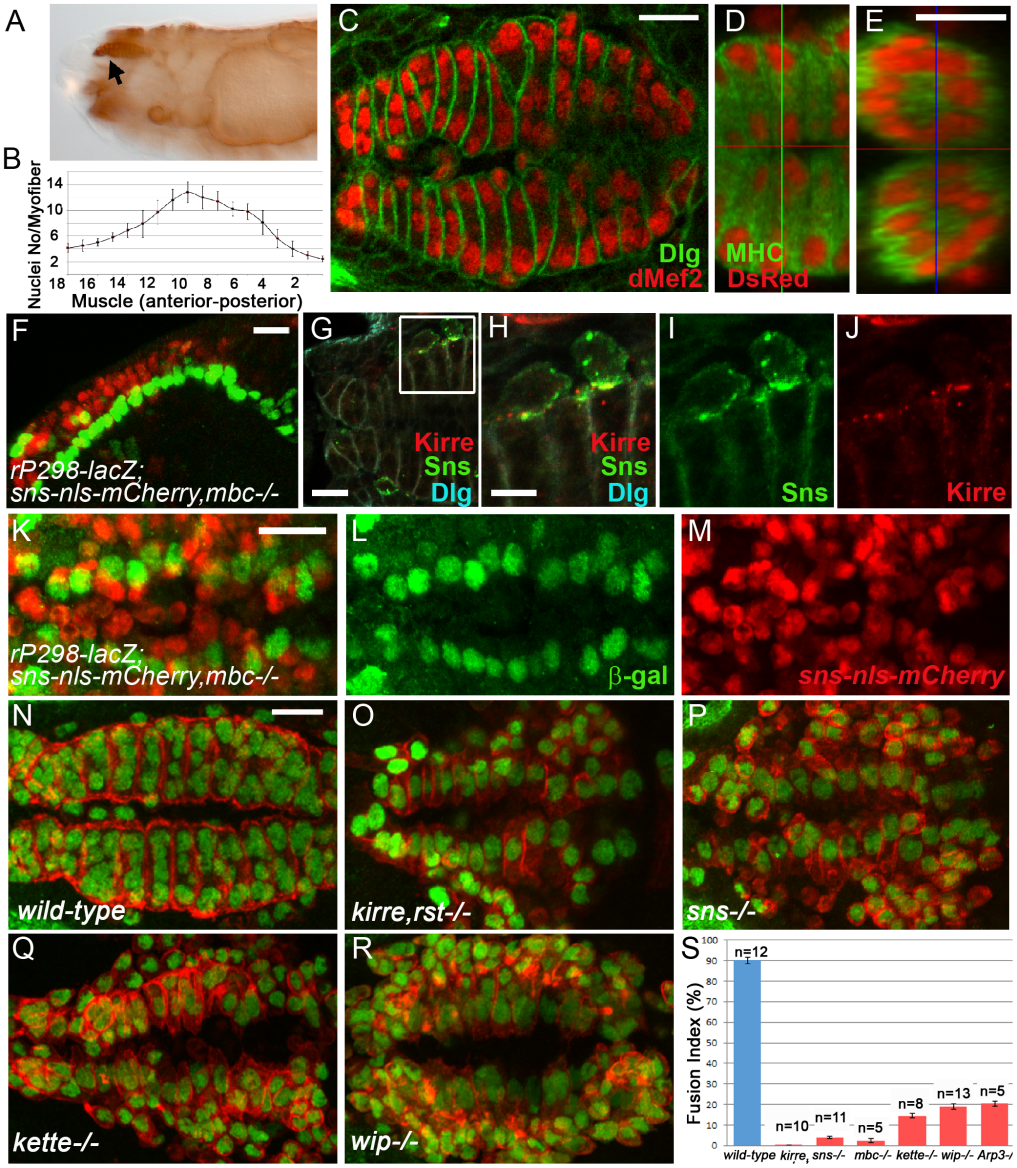
Morphological features of the DPM. (A) Lateral view of a *wild-type* stage 16 embryo showing the position of the pharyngeal muscles (arrow). (B). Quantitation of nuclei in each myotube establishes the number and size of the muscle array in each hemisegment. The error bars denote standard error of the mean. (C) Dorsal view of a representative *wild-type* stage 16 embryo immunostained for dMef2 and Dlg. (D, E) Stage 16 embryo expressing *sns-nls-mCherry* stained for DsRed and endogenous MHC, with orthogonal view in XZ axis in (E) showing the positioned nuclei near the periphery of the myotube. (F, K–M) Confocal projections of stage 15 lateral view (F) and stage 16 dorsal view (K–M) *mbc^D11.2^* embryo expressing FC marker *rP298-lacZ* and FCM marker *sns-nls-mCherry* immunostained for β-gal (green) and DsRed (red) respectively. FCMs are primarily dorsal and lateral to the FCs. (G–J) Stage 15 *wild-type* embryo immunostained for Sns, Kirre and Dlg. Sns and Kirre colocalize at points of FCM-myotube contact. (N–R) Dorsal view of stage 15 embryos immunostained for dMef2 and tropomyosin. Each image is a projection of several confocal Z sections. (S) The Fusion Index reveals significant fusion defects in various mutant embryos compared to that occurring in *wild-type* embryos. The error bars denote standard error of the mean. N denotes number of DPM analyzed. Scale bar: (C–G, K–R) 10 µm, (H–J) 5 µm.

The DPM overlays and controls movement of the pharynx in the *Drosophila* larva, and includes 16–18 tightly associated, bilaterally symmetric muscle fibers (Fig. 1A–C). Since this musculature undergoes less extensive morphogenetic movement than cells in the abdominal segments, it is better suited for visualization of myoblast fusion in living embryos. dMef2 and Dlg reveal the overall pattern of muscles (Fig. 1C), with the number of nuclei in each syncytium ranging from 4 to 14 (Fig. 1B, C). Thus, the size of these muscles is similar to that of the body wall muscles [41]. These nuclei become positioned in a ring around the periphery of the fully formed muscle (Fig. 1D, E). At early stages, the cells that give rise to the DPM are distributed along the dorsoventral axis in layers that curve around the developing pharynx. Posterior and dorsal migration results in a flattening of this array as the muscles increase in size through fusion with FCMs and come close to the surface (Fig. S1C–G).

The DPM forms using the same basic principles as the body wall muscles [14], [42]. Individual myotubes derive from fusion between Kirre-expressing FC and Sns-expressing FCMs, as evidenced by Sns and Kirre at points of FCM:myotube contact as well as distinct populations of cells that express the FC marker rP298-lacZ and the FCM-specific reporter *sns-nls-mCherry* [37] (Fig. 1G–J, Fig. S1A, B). These two distinct cell populations are most apparent in embryos mutant for *mbc*, which are lacking the nonconventional guanine nucleotide exchange factor (GEF) for Rac1, and in which fusion does not occur [27]. Also evident is that the FCMs lie either parallel or dorsal to the myotubes with which they fuse. Quantitation of the mononucleate β-gal positive cells in *mbc* mutant embryos revealed an array of approximately 32 bilaterally symmetric cells in each animal, consistent with one FC for each muscle fiber (Fig. 1F, K–M). Furthermore, embryos deficient for FC-specific adhesion molecules *kirre* and *rst*, mutant for the FCM-specific adhesion molecule *sns* and *mbc* described above, and regulators of branched actin that include *kette*, *wip* and *Arp3,* all show severe fusion defects (Fig. 1N–S, Movies S1–5), as reported for the somatic musculature.

### Time-lapse imaging reveals dynamic membrane protrusions and actin filaments that precede overt myoblast fusion

**Figure 2:**
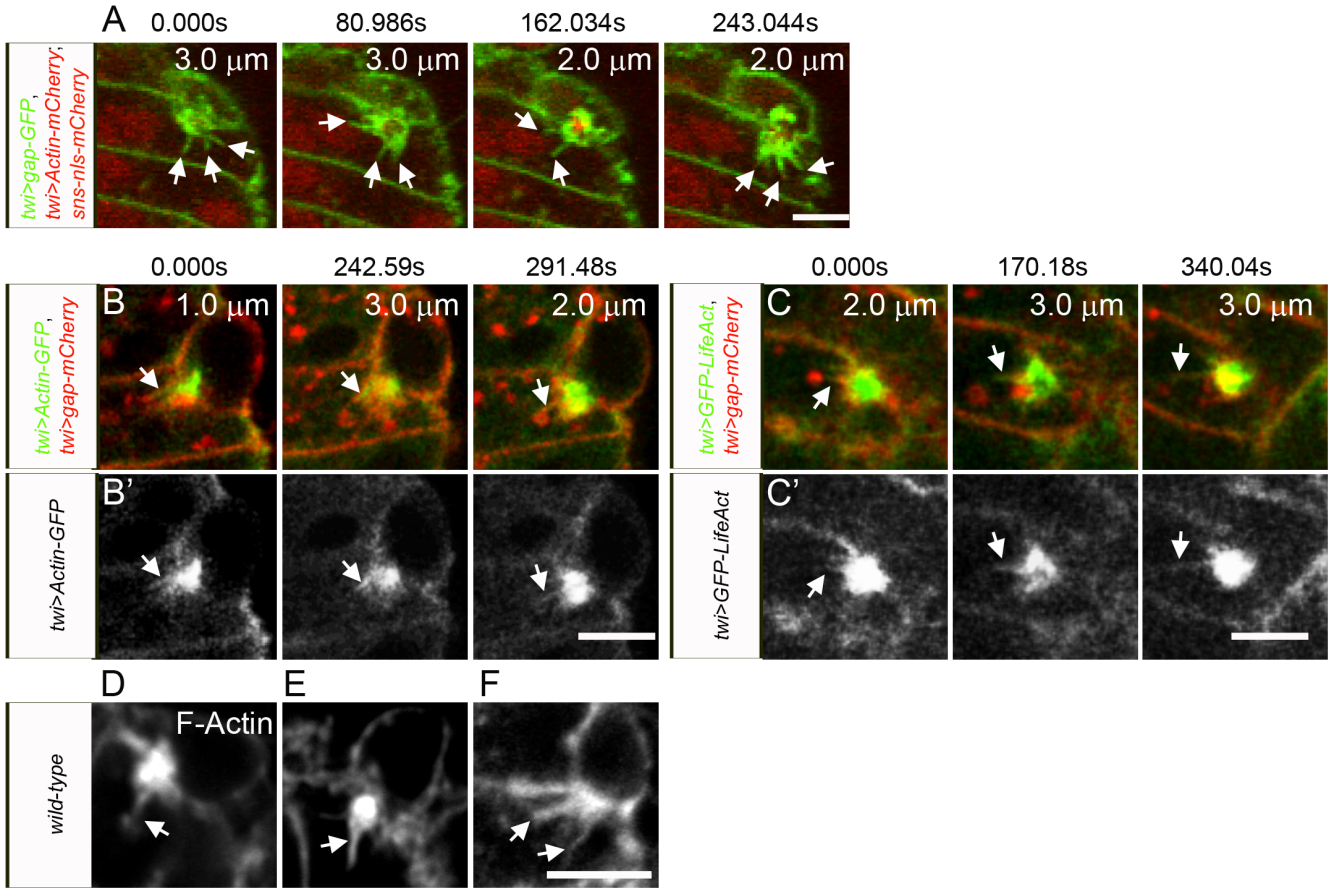
Dynamic actin-containing membrane protrusions from the FCMs into the myotube precede myoblast fusion in the DPM. (A) Time lapse of stage 13 *wild-type* embryo expressing *sns-nls-mCherry*, *twi*>*gap-GFP* and *twi*>*Actin-mCherry*. The mCherry signals were collected in the same channel but note that *sns-nls-cherry* marks the nuclei and *twi>Actin-mCherry* was very weak and is not clearly visible under these conditions. See Fig. S2 and Movie S6 for entire fusion event. Dynamic membrane protrusions are evident prior to fusion. (B) Time lapse of stage 13 *wild-type* embryo expressing *twi*>*gap-mCherry* and *twi*>*Actin-GFP*. (C) Time lapse of stage 13 *wild-type* embryo expressing *twi*>*gap-mCherry* and *twi*>*GFP-LifeAct.* (B′, C′) The actin channels in B and C are shown in grayscale in B′ and C′, respectively, highlighting the presence of actin in filopodial protrusions. (B′′, C′′) High magnification views of B′ and C′ depicting the filopodial actin. (D–F) Phalloidin stained fixed *wild-type* embryos shows F-actin in thin protrusions. Scale bar: 5 µm.

We next visualized individual fusion events in these muscles in live embryos. As noted above, the DPM undergoes less morphogenetic movements during their formation compared to the body wall muscles. Thus, myoblasts remain in the field of view for longer periods of time, shorter time points can be captured since less confocal sections are required, and transient structures are more readily observed. Initially, the membranes of myoblasts and developing muscles were marked by membrane targeted GFP under control of the *twist* promoter (*twi*>*gap-GFP*). FCM and myotube nuclei were visualized by expression of *sns-nls-mCherry*. Figure 2 highlights single fusion events captured with different markers and microscopy conditions. Figure 2A shows a fusion event over approximately nine minutes, using *twi*>*gap-GFP* to mark the membrane, and reveals the presence of dynamic membrane protrusions prior to overt fusion (see also Fig. S2, Movie S6). Notably, the changes in gap-GFP at this single point of contact indicate that the protrusions are not static, extending and retracting as apparent in successive confocal Z sections separated in time by approximately 80 seconds. A concentration of gap-GFP is also apparent at sites of cell contact, consistent with multiple convoluted membrane protrusions at this site.

Additional time-lapse series were carried out using gap-mCherry and either Actin-GFP or GFP-LifeAct under *twi* control. In these studies, the membrane targeting gap sequence fused with monomeric mCherry was used to minimize the potential for oligomer formation and consequent protein aggregation. Actin-GFP is visible as both monomeric and polymerized actin while GFP-LifeAct recognizes only polymerized actin. Actin-GFP and GFP-LifeAct were similar to each other in intensity, and both were more robust and less sensitive to bleaching than Actin-mCherry in our hands. These time series (Fig. 2B, C) were carried out under conditions essentially as above, but with time points every 49 seconds or 170 seconds respectively. In both cases, we observed an intense actin focus in FCMs at sites of contact with developing myotubes as expected (Fig. 2B, C). As previously reported for actin foci in the body wall musculature [2], the average length of time from the initial appearance of an actin focus until overt fusion is approximately 12 minutes, with some actin foci persisting for more than 30 minutes. Importantly, however, this actin focus was accompanied by actin protrusions that extend into the myotube (Fig. 2B–C′) and change over time. Prominent actin staining in the filopodia was also observed in fixed tissues of *wild-type* embryos stained with phalloidin (Fig. 2D–F). While we are unable to assess the transient and dynamic nature of these filopodia, they do not appear to be exclusive to the DPM, and similar structures are present in the body wall musculature in fixed tissues of similarly staged *wild-type* embryos (Fig. S3). We also used spinning disk confocal microscopy on embryos expressing Actin-GFP and gap-mCherry reporters, and allowed examination every 12 seconds (Movie S7). As in the above analysis, changing membrane protrusions are visible. These observations are in some contrast to described by Duan et al. [43], who reported seeing actin filopodia in embryos mutant for type 2 p21-activated kinases but not during normal fusion in *wild-type* embryos. We can only speculate that these were not seen in *wild-type* embryos due to their transient nature and challenge to observe in cells when fusion is not blocked genetically.

### Filopodia and filaments visible at the cell:cell interface by ultrastructural analysis

**Figure 3:**
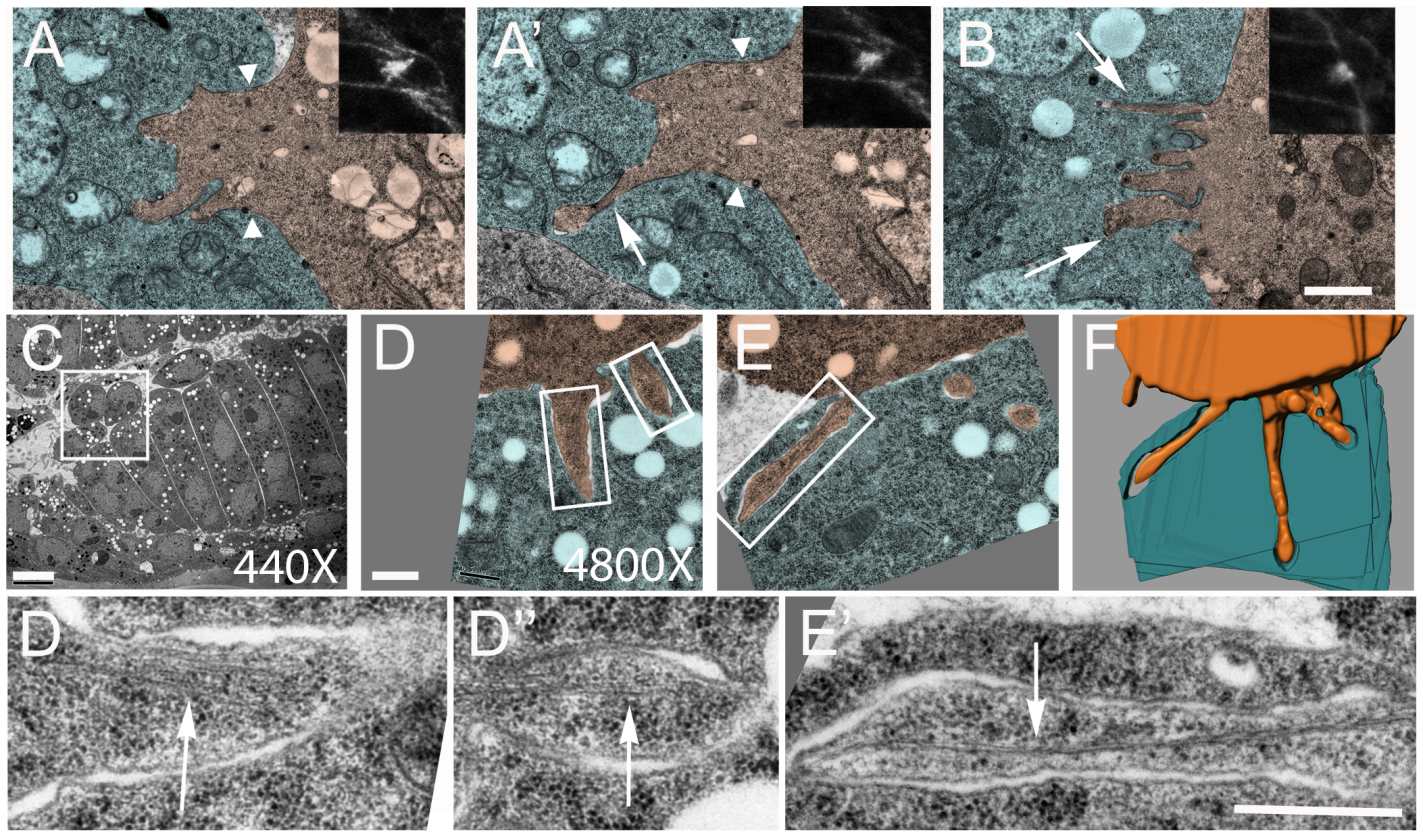
Morphologically distinct protrusions at F-actin foci and thin filaments in fingers. (A–B) Conventional TEM analysis of two actin-rich foci at points of FCM-myotube contact in stage 14 *wild-type* embryos. Insets: Confocal analysis of F-actin foci visualized by phalloidin staining embryos prior to analysis by TEM. (A, A′) Two different sections of the same focus; Note the broad region of invasion marked by arrowheads, with finger-like projections at the distal end (arrows). (B) An FCM projects multiple invasive fingers into a myotube (arrows). (C–E) DPM of stage 14 *wild-type* embryo fixed by HPF-FS. (C) Low magnification view (D, E) High Magnification views of two sections of the FCM boxed in C. (F) 3D reconstruction of the entire serial section (see Fig. S4A–I). (D′–D′′, E′) Higher magnifications of the fingers boxed in D and E reveals multiple linear filaments of ∼7–10 nm in diameter (arrows). Scale bars: A, A′, B: 1 µm, C: 5 µm, D–E′: 0.5 µm. FCMs and myotubes in the DPM are pseudo-colored orange and blue, respectively.

The features of the DPM noted above as advantageous for live imaging also facilitated examination of the subcellular ultrastructure of these actin foci by conventional TEM. We initially used a modified approach in which the location of actin foci was determined in fixed phalloidin-stained embryos by confocal microscopy, and the same embryos were then processed for high resolution imaging by TEM. This analysis clearly demonstrated protrusive structures that emanate from the FCMs into the myotubes at the actin foci (Fig. 3A–B). We observed thin finger-like projections that varied from 150–500 nm in width and from 0.5–2.5 µm in length (Fig. 3B), as previously described in the body wall muscles using HPF-FS [4], [5]. We also observed wide protrusions approximately 2 µm wide and 2.5 µm deep with fewer finger-like projections (Fig. 3A). In addition we detected finger-like projections up to 2.2 µm in length emanating from the end of wide protrusions (Fig. 3A′). The membrane appears to be continuous in these protrusions, suggesting that fusion pores may not have formed yet. This hypothesis is consistent with previous suggestions that actin focus breakdown precedes fusion [2], and the suggestion from other studies that small pores are not visible because they expand quite rapidly [5].

We also examined the FCM:myotube interface in the DPM by HPF-FS, which is incompatible with prior phalloidin staining but better preserves cell structures, and assembled serial sections for visualization in three dimensions (Fig. 3C–F, Fig. S4). Two features distinguish this example from previously published invasive structures. First, we note much longer, apparently independent, fingers that extend into the myotubes in different directions. Second, linear filaments, 7–10 nm in diameter, are at the core of these fingers (Fig. 3D′–E′, Fig. S4L, M). These data are the first to demonstrate the presence of linear filaments at sites of myoblast fusion that most likely coexist with Arp2/3 based branched actin structures at points of fusion. Since immunoEM and HPF-FS is incompatible in our hands, we cannot confirm the molecular nature of these filaments. Nevertheless, their width is inconsistent with microtubules and more consistent with actin filaments. Thus, though all criteria for conventional filopodia have not been confirmed, we refer to these and the previously observed dynamic actin-associated projections as filopodia and filopodia-like projections for simplicity.

### The formin Dia decorates actin filaments, which increase in *Arp3* mutant embryos

**Figure 4:**
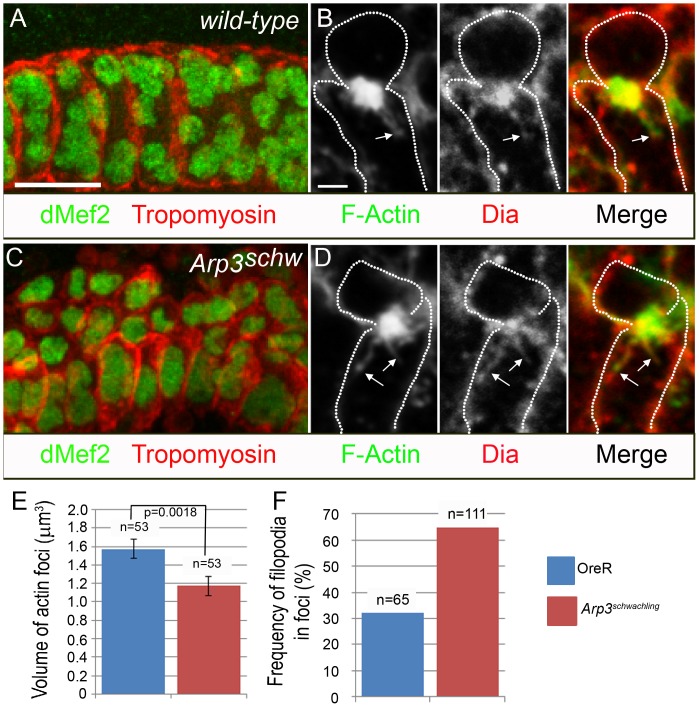
Filopodia formation increases while F-actin volume decreases in the FCM in *Arp3* mutants. (A, C) Stage 16 *wild-type* (A) or *Arp3^schw^* mutant (C) immunostained for dMef2 and tropomyosin. Fusion defects are apparent in *Arp3^schw^* mutant compared to *wild-type*. (B, D). Stage 14 *wild-type* (B) or *Arp3^schw^* mutant (D) stained for F-actin and Dia. The formin Dia is present within the F-actin focus, along the length, and at the tip of F-actin filopodia (arrows) in *wild-type* and *Arp3^schw^* mutants. (E) Quantitation of F-actin volume in *wild-type* and *Arp3^schw^* mutants. Error values are standard error of the mean; n denotes the number of actin-enriched FCMs examined from a total of 16 embryos for *wild-type* and 8 embryos for *Arp3^schw^* mutants. (F) Quantitation of the frequency of foci with filopodia in *wild-type* and *Arp3^schw^* mutants; n is the number of actin-enriched FCMs analyzed from 14 embryos in *wild-type* and 9 embryos from *Arp3^schw^* mutants. Scale bar: (A, C) 10 µm, (B, D) 2 µm.

The presence of dynamic membrane projections and linear filaments clearly suggest the involvement of molecules in addition to those mediating formation of branched actin in myoblast fusion. Formins such as Dia are potent nucleators of linear F-actin filaments and promote their elongation by blocking capping proteins from binding to the barbed end of actin filaments. Interestingly, Dia consistently co-localized with F-actin at the focus and was often observed at discrete points along the length of filopodia-like projections (Fig. 4B). Arp3 is also observed at sites of actin enrichment in the FCM (Fig. S5).

Since Dia, a nucleator of bundled unbranched actin, and Arp3, a nucleator of branched actin, both coexist at actin enrichments in the FCM, it was of interest to see whether these two pathways might be competing for actin and influence the corresponding structures. We first examined *Arp3* mutants for fusion defects and changes in filopodia formation. Though some fusion does occur in these mutant embryos in both the DPM and body wall muscles (Fig. 1S [15]), possibly as a consequence of maternally provided protein, they displayed significant fusion defects (Fig. 4A, C). Careful quantitation in double-blind studies showed that the volume of the F-actin focus decreases in these mutants from 1.57 µm^3^ to 1.17 µm^3^, which might reflect a dependence of the focus on branched actin. Importantly, our analysis also revealed a corresponding increase in the frequency of protrusions emanating from an F-actin focus, from 32.2 percent to 64.8 percent (Fig. 4B, D, E, F). From these data, we hypothesize that the linear and branched actin pathways compete for a limited pool of actin, as described in other systems [22], [44], and that this actin becomes more available in *Arp3* mutants for the formation of the filopodial projections.

### Myoblast fusion and F-actin formation are impaired upon expression of constitutively active Dia

**Figure 5:**
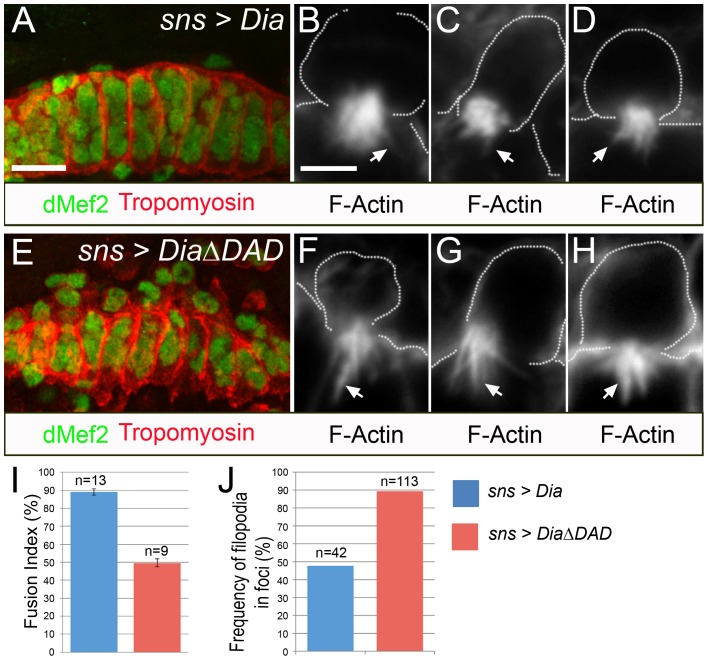
Fusion defects and a reduction in actin foci accompany gain-of-function Dia in the FCMs. (A, E) Stage 16 embryos that are *sns*>*Dia* (A) or *sns*>*DiaΔDAD* (E) immunostained for dMef2 and tropomyosin. Fusion defects are apparent in embryos expressing *sns*>*DiaΔDAD* compared to those expressing *sns*>*Dia.* (B–D, F–H). Three examples of stage 14 embryos expressing *sns*>*Dia* (B–D) or *sns*>*DiaΔDAD* (F–H) stained with phalloidin to visualize F-actin. The F-actin foci in *sns*>*DiaΔDAD* compared to *sns*>*Dia* are not as dense and have more filopodial protrusions (arrows). (I) Calculation of the Fusion Index reveals fusion defects in embryos expressing *sns*>*DiaΔDAD* compared to those expressing *sns*>*Dia*. The error bars denote standard error of the mean. N is the number of DPM analyzed. (J) Quantitation of the frequency of foci with filopodia in embryos expressing *sns>Dia* and embryos expressing sns> *DiaΔDAD*; n is the number of actin-enriched FCMs analyzed. Scale bar: (A, E) 10 µm, (B–D, F–H) 2 µm.

The above results show that defects in formation of Arp3 mediated branched actin correlate with decreased F-actin foci and increased F-actin protrusions, and the co-localization with Dia is consistent with the involvement of formins in this process. We therefore examined whether expression of constitutively active Dia in the DPM impacts myoblast fusion using a constitutively active form of Dia that lacks the C terminal DAD domain which is necessary to keep it in an auto-inhibitory state (Dia-ΔDAD) [28]. Its expression was driven by *sns-Gal4*, which directs expression specifically to the FCMs and is not expressed either panmesodermally or at earlier stages of development. In contrast to expression of *wild-type* Dia, Dia-ΔDAD is clearly associated with defects in myoblast fusion (Fig. 5A, E, I, Movies S8, S9). In addition to expression from the *sns* enhancer, dMef2 expression is detected in both myoblasts and myotubes, suggesting that the cells have differentiated (Fig. 5A, E). From these data, we hypothesize that the response to constitutively active Dia is cell-autonomous rather than resulting from earlier perturbation of the embryo. We next examined if there was a change in F- actin accumulation in the FCM at points of its contact with the myotube. We observed a notable decrease in F-actin foci and prominent actin projections upon expression of Dia-ΔDAD in the FCMs compared to *wild-type* Dia (Fig. 5B–D, F–H). Moreover, the frequency of filopodia doubles upon expression of *sns*>*Dia-ΔDAD* in the FCMs compared to the *sns*>*Dia* control (Fig. 5J). Thus, expression of constitutively active Dia phenocopies the changes in the morphology of F-actin structures visible upon Arp3 loss-of-function.

### Distinct gap-enriched membrane debris accompany overt fusion

**Figure 6:**
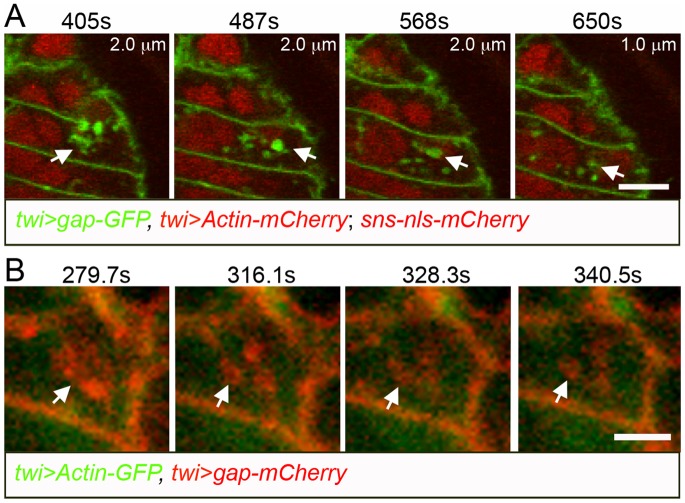
Gap enriched puncta are apparent in the myotube during overt fusion. (A) Time lapse of stage 13 *wild-type* embryo expressing *twi*>*gap-GFP, sns-nls-mCherry*, and *twi*>*Actin-mCherry*. The mCherry signals were collected in the same channel but note that *sns-nls-cherry* marks the nuclei and *twi>Actin-mCherry* was very weak and is not clearly visible under these conditions. See Fig. S2 and Movie S6 for entire fusion event. As the FCM nucleus is absorbed into the myotube, gap-GFP containing puncta are apparent near the site of fusion. (B) Time lapse of stage 13 *wild-type* embryo expressing *twi>gap-mCherry* and *twi*>*Actin-GFP.* See Movie S7 for entire fusion event. Gap-mCherry containing membrane remnants in the myotube are visible near sites of fusion. Arrows mark the membrane-associated puncta. Scale bar: 5 µm.

Our live imaging data of individual myoblast fusion events also revealed that following overt fusion, when the nucleus of the FCM is being absorbed into the myotube, gap-GFP and gap-mCherry become localized in discrete puncta (Fig. 6, Fig. S2, Movie S6, Movie S7). These puncta are visible immediately upon the appearance of membrane discontinuity. The puncta at later time points are observed in centric regions of the cytoplasm, in the middle of the nuclei ring at the myotube periphery. These puncta ultimately disappear, consistent with the trafficking of membrane proteins to other vesicles.

The above data shows that the cell adhesion molecules Kirre and Sns are present in the DPM at discrete points on the surface of myotubes and FCMs, respectively, as previously observed in S2 cells and in the body wall musculature [11], [12]. Moreover, Sns is not detected on the surface of mature myotubes [6].

**Figure 7:**
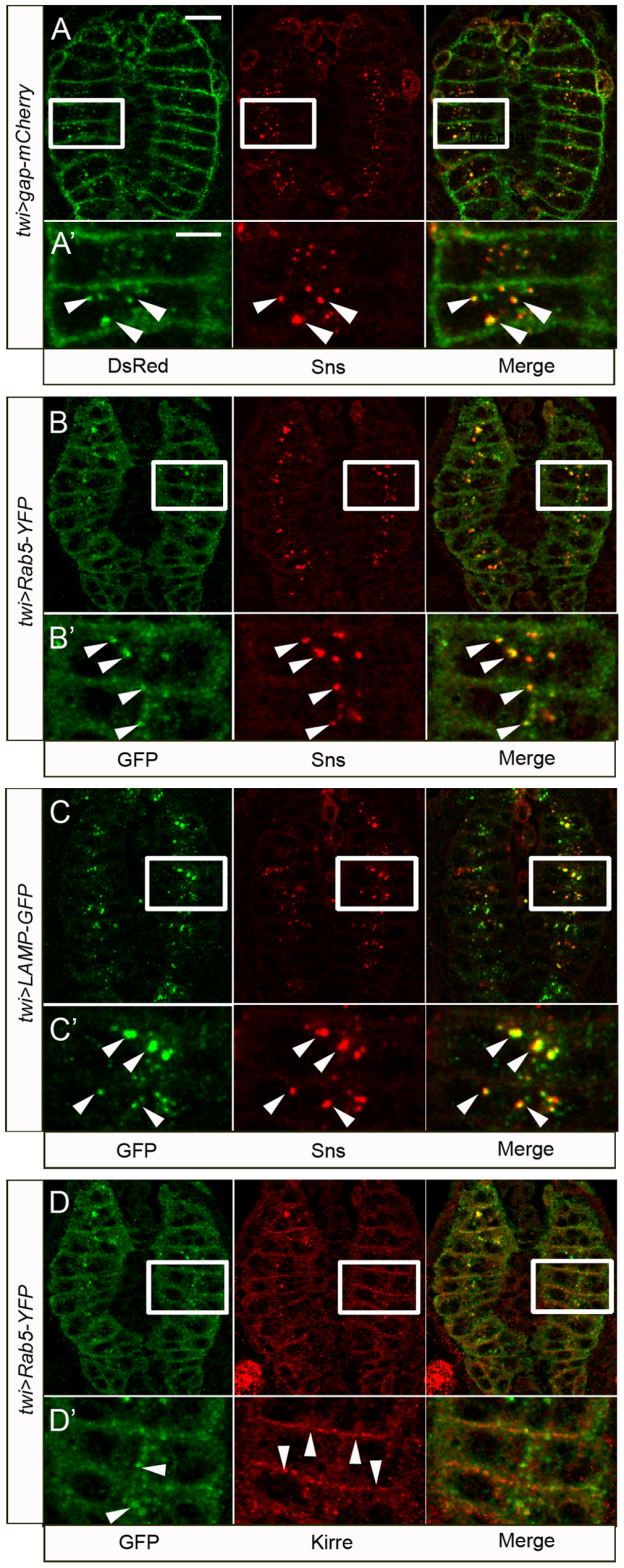
Sns is endocytosed and transported to lysosomes in the myotube following fusion, while Kirre remains associated with the membrane. Late stage 15 *wild-type* embryos expressing *gap-mCherry* (A–A′), *Rab5-YFP* (B–B′, D–D′), or *LAMP-GFP* (C–C′) under *twi-Gal4* control. Embryos are immunostained for the fluorescent reporter and endogenous Sns (A–C′) or Kirre (D–D′). Following fusion, Sns shows prominent colocalization with gap-mCherry (arrowheads in A′), the early endosomal marker Rab5 (arrowheads in B′) or the lysosomal marker LAMP (arrowheads in C′) in the myotube. By contrast, Kirre remains predominantly at the membrane of the myotube (arrowheads in D′) and does not show significant colocalization with Rab5 vesicles. (A′–D′) Higher magnification views of the boxed areas shown in A–D. Scale bar: (A–D) 10 µm, (A′–D′) 5 µm.

To explore the fate of Sns after fusion, in more detail, we examined fixed embryos co-stained for gap-mCherry and endogenous Sns. Sns puncta colocalize with gap-mCherry (Fig. 7A, A′), consistent with the internalization of both membrane proteins upon fusion. We next examined the pattern of Sns in fixed embryos expressing *twi>Rab5-YFP*, a previously described marker for early endosomes [45]. We observed strong colocalization of Sns with endosomes in myotubes (Fig. 7B, B′). As anticipated from this observation, Sns also colocalizes with Rab5 in the larger endosomes of embryos expressing constitutively active (CA) Rab5-YFP (Fig. S6A, A′). By comparison, Sns is more generally dispersed throughout the cytoplasm, as is Rab5, in embryos expressing dominant negative (DN) Rab5-YFP (Fig. S6B, B′). Finally, colocalization of Sns with Rab4 and Rab11 endosomes is very limited (Fig. S6C–D′), and the majority of Sns colocalizes with LAMP-GFP, a marker for lysosomes, consistent with the degradation of Sns in myotubes following fusion (Fig. 7C, C′).

In contrast to Sns, which is unlikely to be needed on the surface of the myotube for its continued association with FCMs, Kirre must be present on the myotube surface to continue attracting FCMs. We therefore examined Kirre to determine whether it exhibited the same puncta as Sns. We observed very little colocalization of Kirre with recycling Rab5-containing endosomes (Fig. 7D, D′) and most of the Kirre levels remain robust at the myotube surface. These data are consistent with an ongoing requirement for Kirre on the myotubes, and suggest that Sns and Kirre are differentially trafficked upon fusion.

We also examined the actin nucleation promoting factors SCAR and WASp, which are enriched in FCMs at its contact site with a FC or myotube in the body wall musculature [2], [5], [15], [16], [19]. Unlike Sns, neither SCAR nor WASp appeared to co-localize with LAMP-GFP (Fig. S7) and were instead found closer to the myotube surface.

## Discussion

### The DPM as a model for myogenesis and myoblast fusion

This study has demonstrated the value of the developing larval DPM as a model for myogenesis and, through live imaging in this tissue, has provided fundamental new insights into the process of myoblast fusion. The DPMs clearly derive from Kirre-expressing FCs and Sns-expressing FCMs, with the proteins co-localizing at points of FCM-myotube contact. In addition, proteins associated with actin polymerization such as WASp, SCAR and Arp3 are enriched at sites of fusion. Consistent with the regulation of F-actin by these genes, we find F-actin foci in the FCM at its contact with the myotube. Previous studies noted fusion defects in *rols* mutants [46] in the DPM, and our current studies extend this to embryos doubly mutant for *kirre* and *rst*, *sns*, *kette*, *Vrp1/wip*, *mbc* and *Arp3*. The nuclei become highly positioned following fusion, residing in a ring around the periphery of the myotube.

Like the myotubes of the body wall muscles, the individual myotubes of the DPM appear to be generated by the fusion of a single FC with multiple FCMs. This conclusion is based on the observation that approximately 32 cells express the rP298-lacZ FC marker in the absence of fusion, and approximately 32 muscles develop in *wild-type* embryos. However, whereas the unique identity of each body wall muscle is dictated by expression of a constellation of identity Transcription Factors (iTFs) in its FC [47], [48], there is currently no evidence to suggest that each of the DPMs possesses a unique identity. Although these muscles differ from each other in size, they have similar orientations and shapes reminiscent of the bundles of myotubes that make up many adult muscles [49], [50]. It is therefore a formal possibility that, FCs giving rise to the DPM may be coordinately specified by a common mechanism. Consistent with this possibility, transcription factors such as Tinman and Zfh-1 are present in all of the DPMs [51].

With regard to the fusion process itself, the limited morphogenetic movements of these muscles along with the use of membrane-targeted reporters has facilitated visualization of membrane dynamics at the fusion interface, and has provided insights into the process of fusion itself. As in the body wall muscles [4], [5], we can visualize asymmetrically localized actin foci in the FCM and finger-like protrusions from the FCM into the myotube. The data described herein expands our understanding of this process, demonstrating the highly dynamic nature of the projections and associated F-actin prior to a fusion event. We also observe a dramatic disintegration of the intervening membrane at sites of fusion that is inconsistent with expansion of a single pore, as occurs in fusion of the hypodermal cells of *Caenorhabditis elegans* [52]. Since “micro” pores have not been observed at the ultrastructural level in any study, presumably due to its rapid expansion to a 300 nm “macro” pore, we can only speculate about the sequence of events at the fusion membrane at this time. Nevertheless, it appears to be dynamic and more complex than previously appreciated.

### Dynamic filopodia-like F-actin protrusions precede the fusion process

Previous studies have revealed the formation and disappearance of F-actin foci at sites of fusion in the developing body wall muscles over time [2]. Studies have also noted thin actin-rich protrusions in the body wall muscles of mutant embryos in which fusion is blocked by loss of group 1 p21-activated kinases, but these structures were not observed in fusing *wild-type* myoblasts [43]. However, such structures are apparent in fusing *wild-type* cells in our analysis. Moreover, we find these structures to be highly dynamic, in contrast to the more stable positions seen in mutant embryos in the previous study. Interestingly, a recent study has also reported the dynamic nature of filopodia [53] while studying for the role of PIP2 in myoblast fusion.

In *wild-type* myoblasts, thin actin-associated membrane protrusions repeatedly extend and retract at a single contact site within tens of seconds prior to myoblast fusion. While methods are not available to establish the molecular identity of filaments visualized by HPF-FS that extend along the length of the protrusions, it is tempting to speculate that these represent linear actin filaments. It is noteworthy that the formin Dia decorates thin F-actin containing protrusions in fixed tissue that are reminiscent of the dynamic actin filaments visible in living embryos. Dia was also detected at the cell cortex, and is frequently enriched near the F-actin foci at the site of adhesion. Formins such as Dia are required for both the formation and maintenance of unbranched actin filaments present in the filopodia [22], [54]. Complicating functional analyses to determine whether this protein is directly involved in fusion, Dia is provided to the egg maternally and is required for earlier events in embryogenesis. To date, myoblast-directed expression of RNAis and dominant negative proteins have been unsuccessful in reducing this gene product.

### F-actin filopodia and F-actin foci may compete for a limiting pool of actin

The potential role of the above filopodia, and whether they depend on polymerization of branched or linear actin, remains to be determined. It is possible that they might represent formin-associated bundles of actin filaments that co-exist with foci containing branched actin. Such an organization is reminiscent of the filopodia and lamellipodia that coexist at the ends of growth cones [55], [56], and recent models have hypothesized the presence of both branched and bundled actin in myoblast fusion [4], [43]. Arp3 and components of both the WAVE/SCAR and Vrp1/WASp complexes, which promote nucleation of branched actin by activation of Arp2/3, are required for myoblast fusion [2], [15], [16], [17], [18], [19], [20]. Multiple studies have now identified podosome-like structures (PLSs) and actin-associated “dimples” that invade the myotube [3], [5] and it has been suggested that short stiff filaments generated by actin branching are crucial to these invasive structures [4], [43].

Interestingly, we find that decreasing branched actin formation in an *Arp3* mutant causes a decrease in the volume of the F-actin foci and a doubling in the number of F-actin filopodia. The modest, but statistically significant decrease in volume of the F-actin focus is consistent with the observation that some fusion still occurs in this mutant [15], likely due to persistence of a maternally provided *Arp3* gene product. We also found that expression of constitutively active Dia exclusively in fusing FCMs caused longer filopodia to be present.

Several other studies have shown an increase in filopodia upon loss of branched actin nucleation [57], [58]. In one final study, knockdown of WAVE was found to reduce lamellipodia and increase filopodia, a phenotype that was recapitulated by expression of constitutively active Dia and blocked by Dia inhibition [58]. A possible mechanism has been suggested for this behavior in which actin and formins are constant and convert the branched network into filopodia when formation of branched actin is compromised and more actin monomers becomes available [22], [44].

### Endocytosis and differential trafficking of cell adhesion molecules: a mechanism to ensure directionality of fusion?

Along with live imaging, the organized alignment of myotubes within the DPM has been particularly useful in revealing endocytic events that accompany myoblast fusion. Discrete puncta of membrane-associated proteins appear over time coincident with overt fusion, and colocalize with endogenous Sns in fixed tissue. Based on the colocalization of Sns with Rab5-containing endosomes, these puncta appear to reflect active endocytosis at points of fusion. Interestingly, genetic interaction studies with the cell adhesion molecule N-Cadherin suggest that it is also removed from the site of fusion between the FC and FCM via endocytosis, in this case mediated by the GEF, Loner/Schizo [59]. Ultimately, Sns accumulates in LAMP-containing lysosomes in the central cytoplasmic region of the myotube and eventually disappears. By contrast, Kirre remains prominent on the surface of the myotubes and does not colocalize significantly with early endosomes marked by Rab5, possibly reflecting that it is not targeted to the endocytic pathway. Thus, in addition to the recruitment of endocytic vesicles to points of fusion, these vesicles are responsible for differential trafficking of Sns and Kirre. This differential degradation may be one way of preventing myotube:myotube fusion after absorption of the FCM fusion machinery into the myotube, and help to ensure the cell-type specific asymmetry that is critical to the fusion process. In agreement with our observation that Kirre remains on the myotube surface, studies have shown that Rols/Ants stabilizes Kirre at the cell surface [60]. These mechanisms will ensure the availability of Kirre for recognition by additional Sns-associated FCMs upon continued fusion. One might also anticipate that retention of Sns-associated fusion machinery could be deleterious and that the myotube therefore degrades some of these proteins.

Interestingly, components of the endocytic pathway play a role in fusion in murine myoblasts [61], [62] though the relationship of this and the endosomes we describe herein remain to be investigated.

## Supporting Information

Figure S1 The DPM moves dorsally and posteriorly as fusion proceeds. (A) Lateral view of an early stage 13 *wild-type* embryo immunostained colorimetrically for Sns to visualize the FCMs. (B) Lateral view of a stage 15 *rP298-lacZ* expressing *mbc^D11.2^* embryo immunostained colorimetrically for β-gal. Note the distribution of FCs and FCMs. (C, D) Lateral views of *wild-type* early stage 13 (B) or late stage 16 (D) embryos in which nuclei of FCs and resulting myotubes are marked by expression of *rP298-lacZ* (red). This view illustrates the curvature of the cell layer and its flattening upon posterior tissue migration. (E–G) Iso-surfacing in Imaris of select time points from a time-lapse confocal analysis of formation of the pharyngeal myotubes viewed dorsally. Iso-surfacing was done manually using the gap-GFP channel as a guide. Nuclei are marked by *sns-nls-mCherry* and membranes by *twi-Gal4>gap-GFP*. Scale bars: (A–D) 10 µm.
(TIF)

Figure S2 Expanded Z-series dataset from Fig. 2A. The complete Z-series for each time point is shown. Stage 13 embryo expressing *gap-GFP* and *Actin-mCherry* under *twi-Gal4* control and *sns-nls-mCherry.* As previously noted, the mCherry signals were collected in the same channel but *sns-nls-cherry* marks the nuclei and *twi>Actin-mCherry* was very under these conditions. Progression through time is from left to right while progression in Z is from top to bottom. Scale bar: 5 µm.
(EPS)

Figure S3 Actin filopodia visible at the myotube:FCM interface in the body wall musculature of *wild-type* embryos. (A–B) Lateral view of stage 14 *wild-type* embryos stained for phalloidin (F-actin) and immunostained for tropomyosin to visualize actin-containing filopodia that project from the FCM into the myotube in the body wall musculature. (A′–B′). High magnification view of the boxed region in A and B. Scale bar: 5 µm.
(TIF)

Figure S4 TEM by HPF/FS of two DPMs. (A–I) Expanded serial sections from dataset in Fig. 3D–E. Sections are ∼80 nm apart. (J–L) DPM of a *wild-type* stage 14 embryo fixed for TEM by HPF/FS. (K) High Magnification view of a section of the FCM boxed in J. (L, M) Higher magnification of two serial sections, ∼80 nm apart of the finger boxed in K reveals linear filaments of ∼7–10 nm in diameter (arrows). FCMs and myotubes in the DPM are pseudo-colored orange and blue, respectively. Scale bars: A–I, K: 0.5 µm, J: 2 µm, L, M: 0.2 µm.
(TIF)

Figure S5 The branched actin nucleator Arp3 localize to actin foci in the FCM. Visualization of Arp3 and F-actin foci by phalloidin staining in the DPM of stage 14 *wild-type* embryos. The branched actin nucleator, Arp3 (denoted by arrow) is prominent in F-actin focus of *wild-type* myoblasts. Scale bar: 2 µm.
(TIF)

Figure S6 Sns localization in the myotube is affected by Rab5 activity in early endosomes, and traffics to recycling endosomes. Late stage 15 *wild-type* embryos expressing *CA-Rab5-YFP* (A, A′), *DN-Rab5-YFP* (B, B′), *Rab4-YFP* (C, C′) or *Rab11-YFP* (D, D′) under *twi-Gal4* control. All embryos are immunostained for GFP and Sns. (A, A′) Sns is present in large endosomes formed by expression of CA-Rab5. (B–B′) Disrupting the integrity of early endosomes by expressing DN-Rab5 led to mislocalization of Sns to the myotube membrane (arrows in B′). (C–C′) Sns shows prominent colocalization with fast recycling endosomes marked by Rab4 (arrows in C′), and with slow recycling endosomes marked by Rab11 (arrows in D′). A′–D′ are high magnification views of the boxed area in A–D. Scale bar: (A–D) 10 µm, (A′–D′) 5 µm.
(TIF)

Figure S7 Actin nucleation promoting factors SCAR and WASp are not degraded post-fusion and localize to the myotube membrane. (A–B′) Late stage 15 *wild-type* embryos expressing *twi*>*LAMP-GFP*. Embryos are immunostained for GFP and endogenous SCAR (A–A′) or WASp (B–B′). WASp and SCAR are enriched at point of FCM:myotube contact (arrows). (A′, B′) Higher magnification views of the boxed areas shown in A, B. SCAR and WASp are predominantly at the membrane of the myotube (arrowheads) and are not prominent in lysosomes marked with LAMP-GFP. Scale bar: (A, B) 10 µm, (A′, B′) 5 µm.
(TIF)

Movie S1 3D movie of the stage 15 *sns^XB3^* embryo shown in Fig. 1P, highlighting the small myotubes and large number of unfused myoblasts. The embryo was immunostained with dMef2 to monitor the nuclei (green) and tropomyosin to mark the membrane (red), and data processed as described in Materials and Methods. Transparency of the isosurface was adjusted for visualization of nuclei within myotubes.
(MOV)

Movie S2 3D movie of the stage 15 *kette^J4^* ^–48^ embryo shown in Fig. 1Q, highlighting the small myotubes and large number of unfused myoblasts. The embryo was immunostained with dMef2 to monitor the nuclei (green) and tropomyosin to mark the membrane (red), and data processed as for Movie S1 and described in Materials and Methods.
(MOV)

Movie S3 3D movie of the stage 15 *wip^D30^* embryo shown in Fig. 1R, highlighting the small myotubes and large number of unfused myoblasts. The embryo was immunostained with dMef2 to monitor the nuclei (green) and tropomyosin to mark the membrane (red), and data processed as for Movie S1 and described in Materials and Methods.
(MOV)

Movie S4 3D movie of the stage 15 *Arp3^schwachling^* embryo shown in Fig. 4C, highlighting the small myotubes and large number of unfused myoblasts. The embryo was immunostained with dMef2 to monitor the nuclei (green) and tropomyosin to mark the membrane (red), and data processed as for Movie S1 and described in Materials and Methods.
(MOV)

Movie S5 3D movie of the stage 15 *wild-type* embryo shown in Fig. 1N, to emphasize the large myotubes and small number of unfused myoblasts compared to mutant embryos. The embryo was immunostained with dMef2 to monitor the nuclei (green) and tropomyosin to mark the membrane (red), and data processed as for Movie S1 and described in Materials and Methods.
(MOV)

Movie S6 3D movie of the fusion event depicted in Fig. 2A, S2, with only the *twi>gap-GFP* signal. Each time point includes three successive Z sections. The movie was generated using Imaris and Image J. Isosurfacing was done as described in Material and Methods, snapshots taken in Imaris and then aligned and stitched together using the concatenate function in ImageJ. Scale bar: 2 µm.
(AVI)

Movie S7 A fusion event in the DPM of a stage 13 live embryo expressing *twi*>*gap-mCherry* and *twi*>*Actin-GFP.* Confoccal images were collected using the spinning disk microscope and processed as described in Material and Methods. Time between frames is ∼12 seconds. Asterisk marks the FCM that will fuse to the myotube; filopodia from the FCM are denoted with an arrow and gap-enriched membrane debris in the myotube by an arrowhead. Scale bar: 5 µm.
(AVI)

Movie S8 3D movie of the stage 15 embryo expressing *sns>Dia* shown in Fig. 5A, immunostained with dMef2 to monitor the nuclei (green) and tropomyosin to mark the membrane (red). Isosurfacing in Imaris highlight the large myotubes and few unfused myoblasts compared to embryo expressing *sns>DiaΔDAD* (Movie S9) and was generated in a similar manner as for Movie S1.
(MOV)

Movie S9 3D movie of the stage 15 embryo expressing *sns>DiaΔDAD* shown in Fig. 5E to highlight the unfused myoblasts. The embryo was immunostained with dMef2 to monitor the nuclei (green) and tropomyosin to mark the membrane (red), and data processed as for Movie S1 and described in Materials and Methods.
(MOV)
