# Supplementary figures and images for: Live Imaging Provides New Insights on Dynamic F-Actin Filopodia and Differential Endocytosis during Myoblast Fusion in Drosophila
Source: PLoS One. 2014 Dec 4;9(12):e114126. doi: 10.1371/journal.pone.0114126 (PMC4256407; doi:10.1371/journal.pone.0114126)

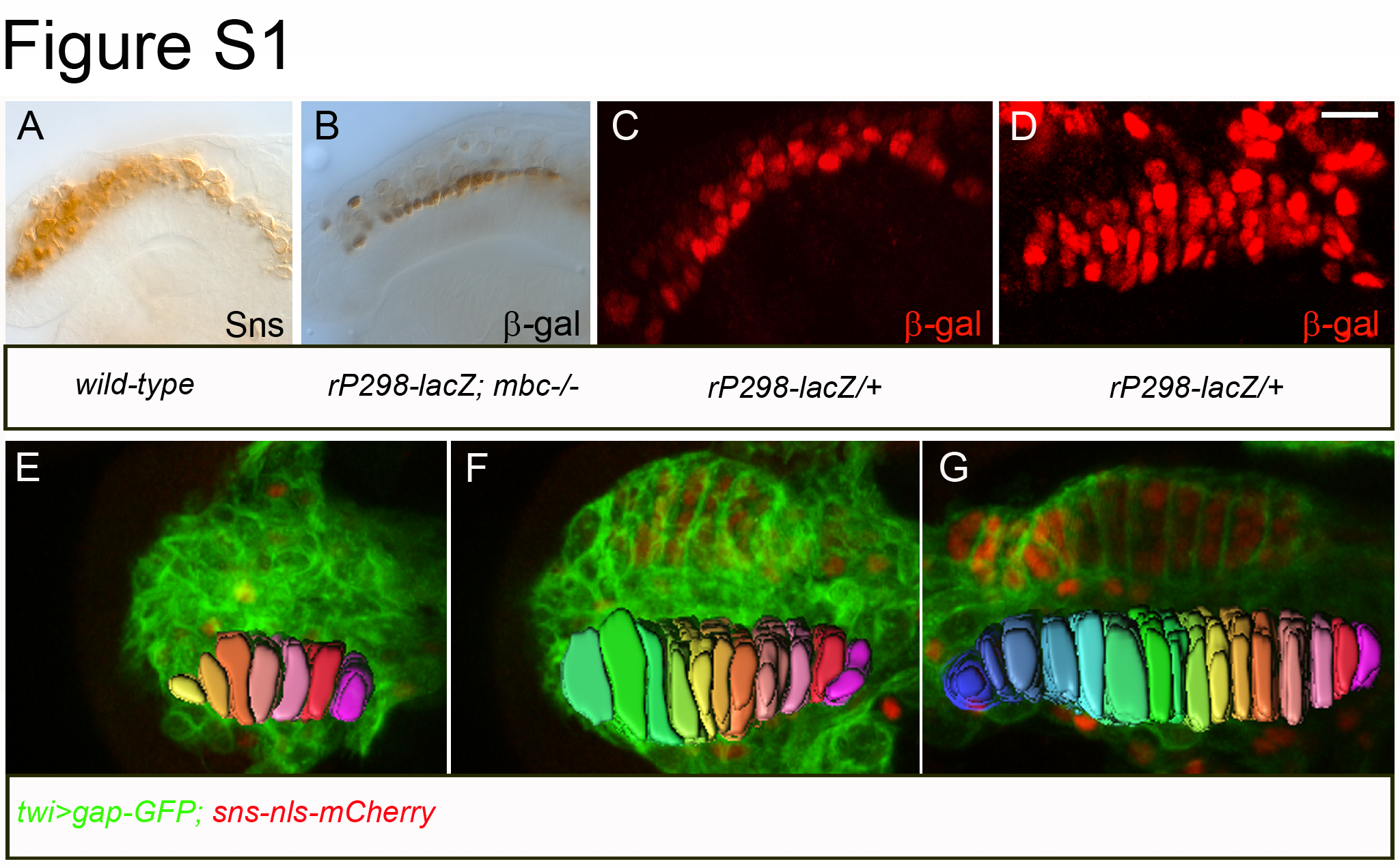

Supplement: Figure S1 — The DPM moves dorsally and posteriorly as fusion proceeds. (A) Lateral view of an early stage 13 wild-type embryo immunostained colorimetrically for Sns to visualize the FCMs. (B) Lateral view of a stage 15 rP298-lacZ expressing mbcD11.2 embryo immunostained colorimetrically for β-gal. Note the distribution of FCs and FCMs. (C, D) Lateral views of wild-type early stage 13 (B) or late stage 16 (D) embryos in which nuclei of FCs and resulting myotubes are marked by expression of rP298-lacZ (red). This view illustrates the curvature of the cell layer and its flattening upon posterior tissue migration. (E–G) Iso-surfacing in Imaris of select time points from a time-lapse confocal analysis of formation of the pharyngeal myotubes viewed dorsally. Iso-surfacing was done manually using the gap-GFP channel as a guide. Nuclei are marked by sns-nls-mCherry and membranes by twi-Gal4>gap-GFP. Scale bars: (A–D) 10 µm. (TIF) [file pone.0114126.s001.tif]

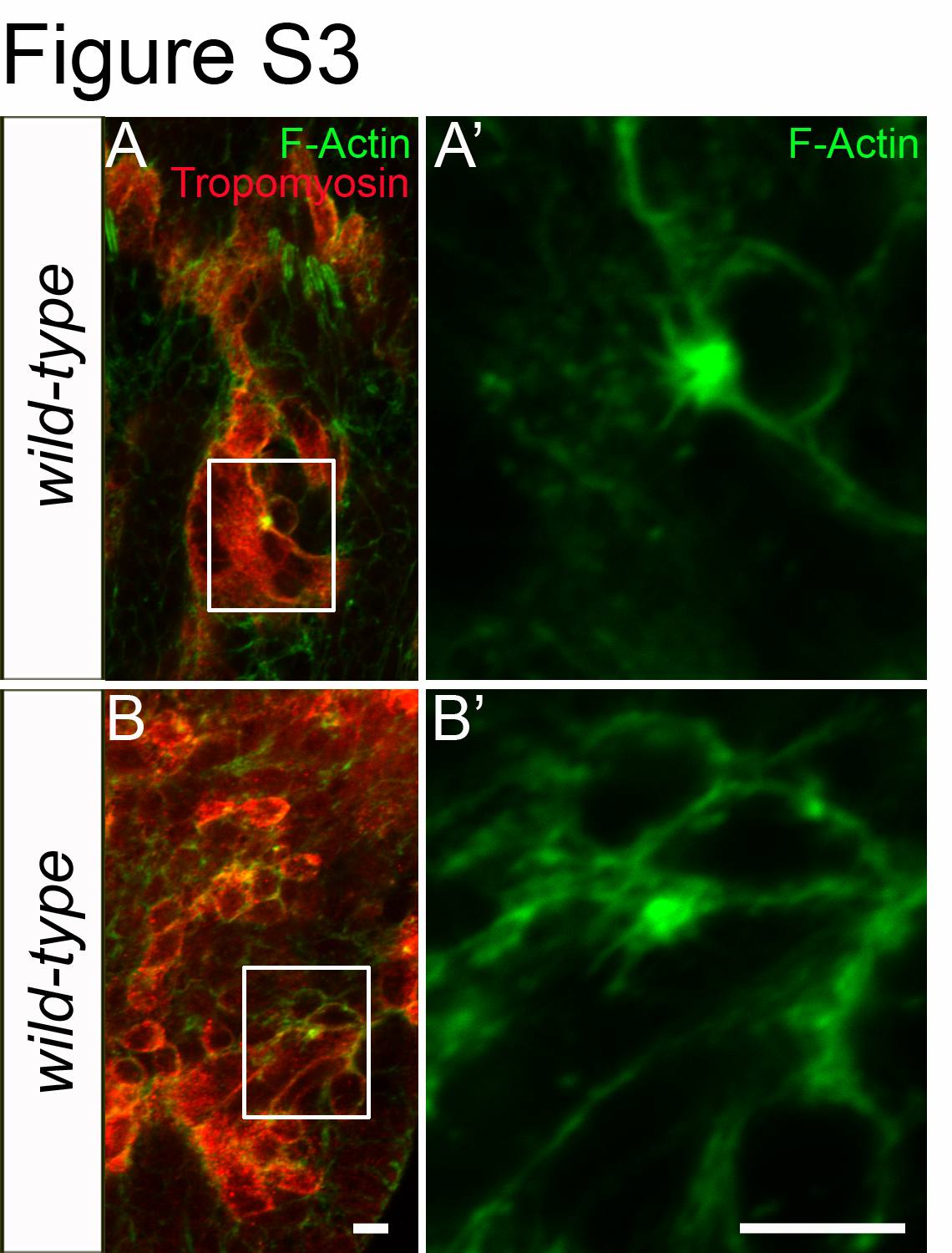

Supplement: Figure S3 — Actin filopodia visible at the myotube:FCM interface in the body wall musculature of wild-type embryos. (A–B) Lateral view of stage 14 wild-type embryos stained for phalloidin (F-actin) and immunostained for tropomyosin to visualize actin-containing filopodia that project from the FCM into the myotube in the body wall musculature. (A′–B′). High magnification view of the boxed region in A and B. Scale bar: 5 µm. (TIF) [file pone.0114126.s003.tif]

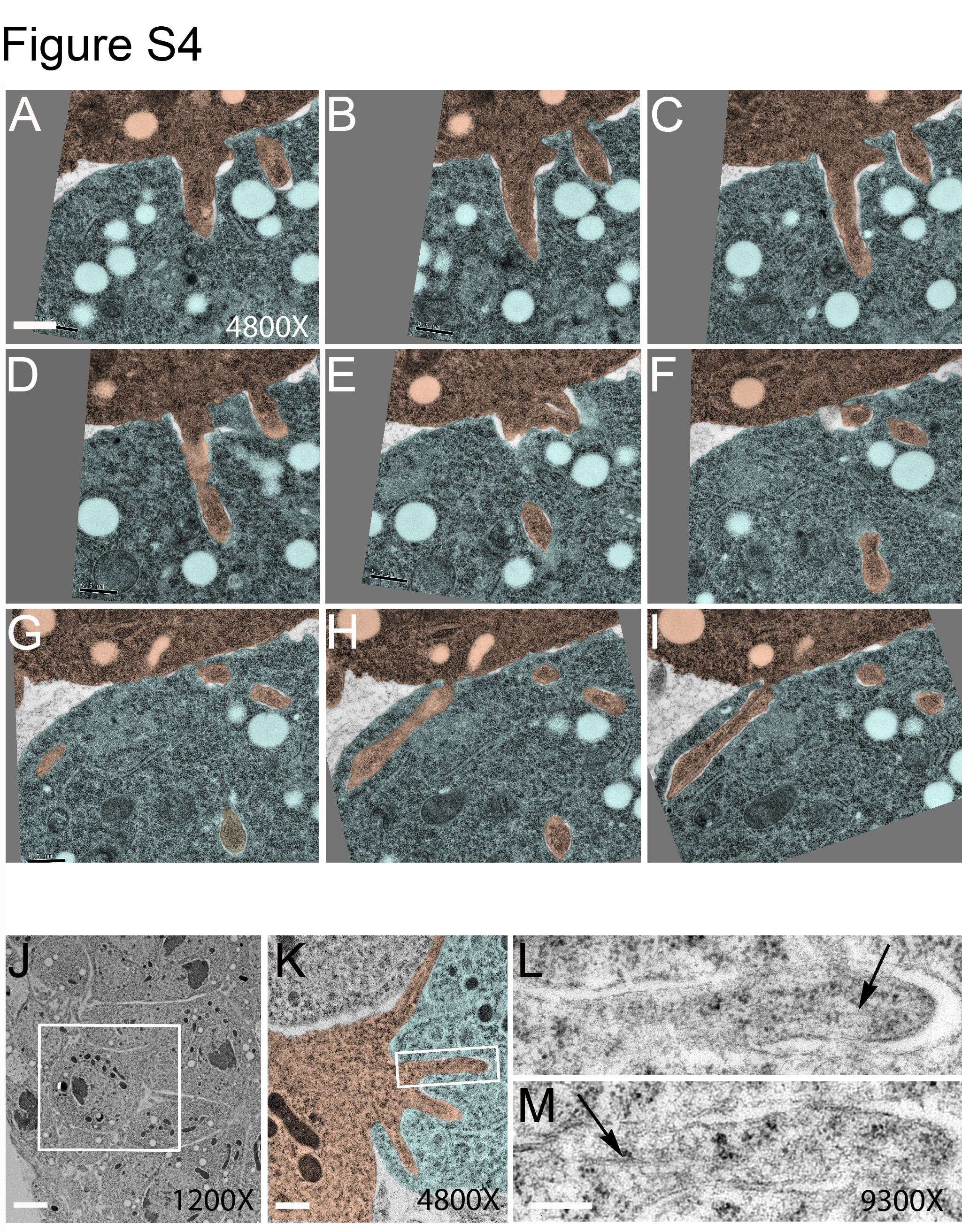

Supplement: Figure S4 — TEM by HPF/FS of two DPMs. (A–I) Expanded serial sections from dataset in Fig. 3D–E. Sections are ∼80 nm apart. (J–L) DPM of a wild-type stage 14 embryo fixed for TEM by HPF/FS. (K) High Magnification view of a section of the FCM boxed in J. (L, M) Higher magnification of two serial sections, ∼80 nm apart of the finger boxed in K reveals linear filaments of ∼7–10 nm in diameter (arrows). FCMs and myotubes in the DPM are pseudo-colored orange and blue, respectively. Scale bars: A–I, K: 0.5 µm, J: 2 µm, L, M: 0.2 µm. (TIF) [file pone.0114126.s004.tif]

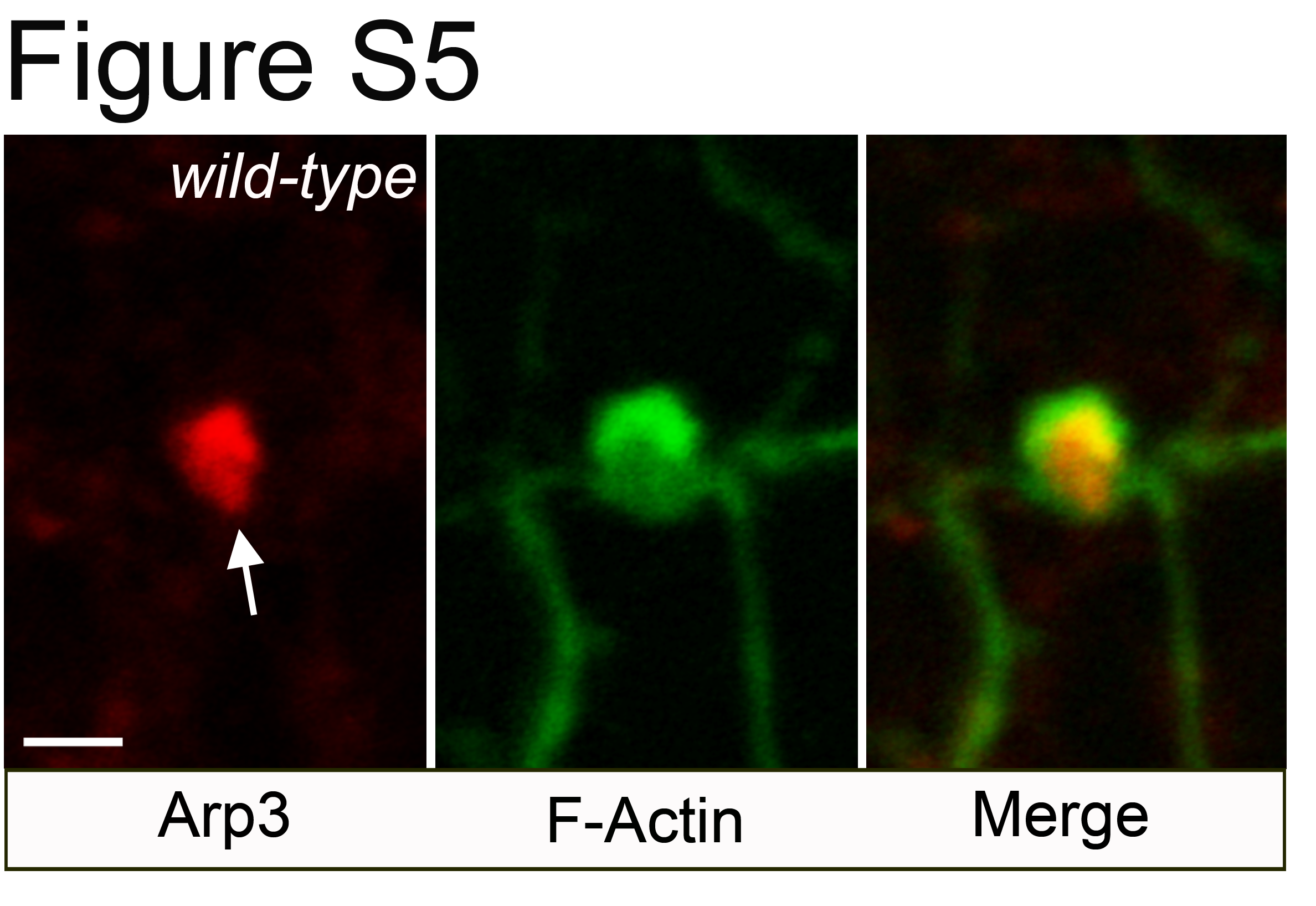

Supplement: Figure S5 — The branched actin nucleator Arp3 localize to actin foci in the FCM. Visualization of Arp3 and F-actin foci by phalloidin staining in the DPM of stage 14 wild-type embryos. The branched actin nucleator, Arp3 (denoted by arrow) is prominent in F-actin focus of wild-type myoblasts. Scale bar: 2 µm. (TIF) [file pone.0114126.s005.tif]

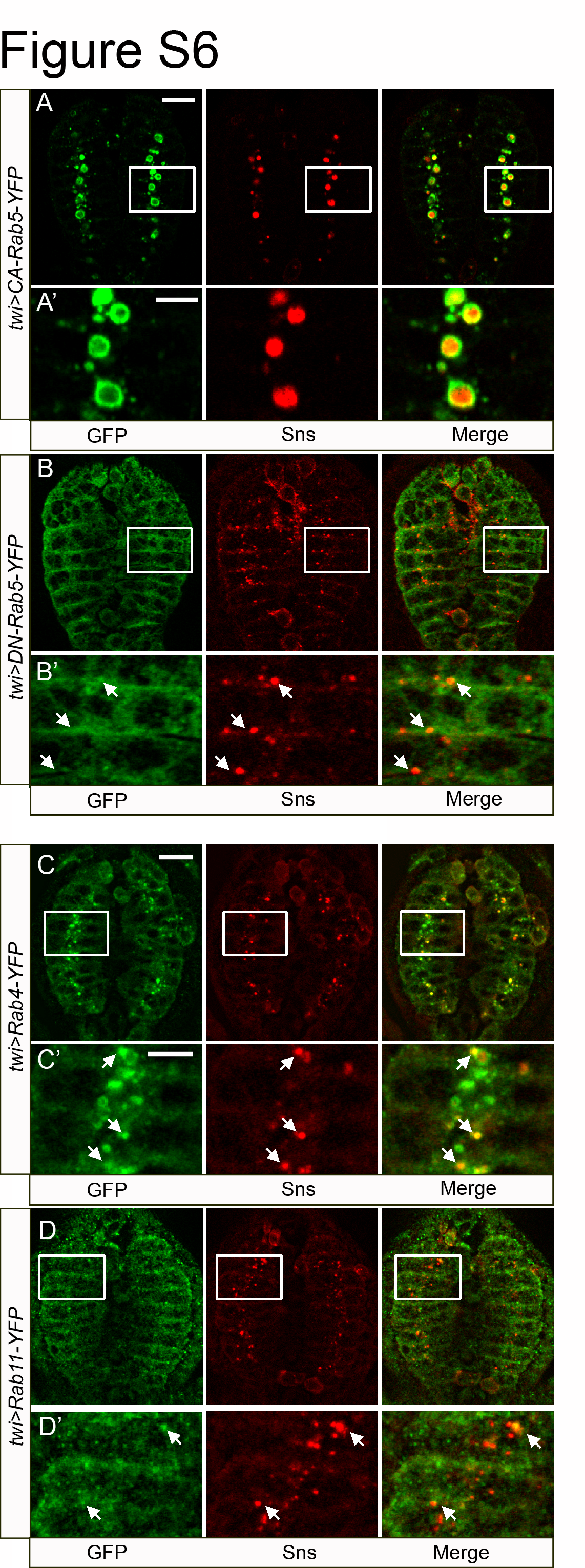

Supplement: Figure S6 — Sns localization in the myotube is affected by Rab5 activity in early endosomes, and traffics to recycling endosomes. Late stage 15 wild-type embryos expressing CA-Rab5-YFP (A, A′), DN-Rab5-YFP (B, B′), Rab4-YFP (C, C′) or Rab11-YFP (D, D′) under twi-Gal4 control. All embryos are immunostained for GFP and Sns. (A, A′) Sns is present in large endosomes formed by expression of CA-Rab5. (B–B′) Disrupting the integrity of early endosomes by expressing DN-Rab5 led to mislocalization of Sns to the myotube membrane (arrows in B′). (C–C′) Sns shows prominent colocalization with fast recycling endosomes marked by Rab4 (arrows in C′), and with slow recycling endosomes marked by Rab11 (arrows in D′). A′–D′ are high magnification views of the boxed area in A–D. Scale bar: (A–D) 10 µm, (A′–D′) 5 µm. (TIF) [file pone.0114126.s006.tif]

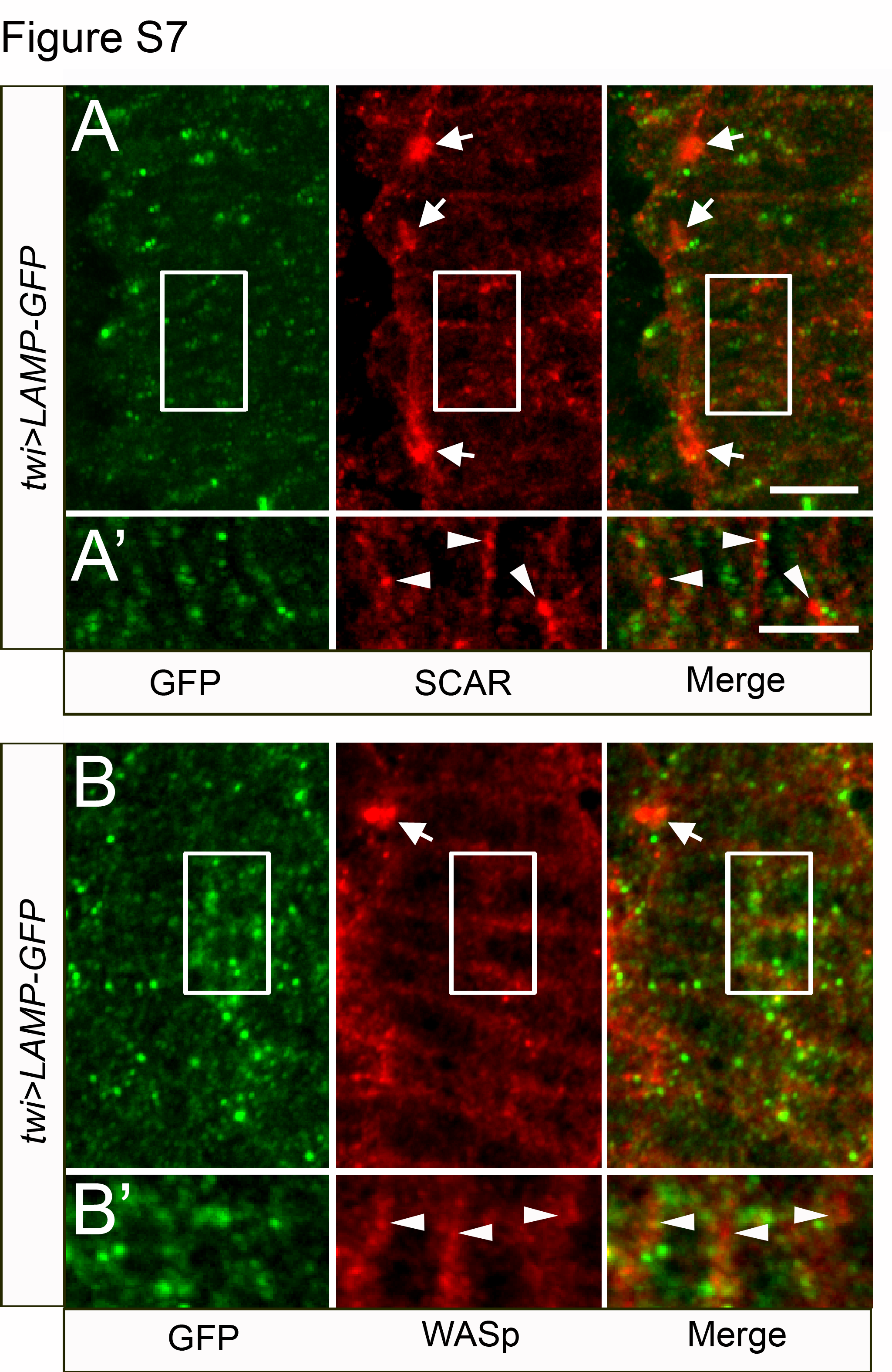

Supplement: Figure S7 — Actin nucleation promoting factors SCAR and WASp are not degraded post-fusion and localize to the myotube membrane. (A–B′) Late stage 15 wild-type embryos expressing twi>LAMP-GFP. Embryos are immunostained for GFP and endogenous SCAR (A–A′) or WASp (B–B′). WASp and SCAR are enriched at point of FCM:myotube contact (arrows). (A′, B′) Higher magnification views of the boxed areas shown in A, B. SCAR and WASp are predominantly at the membrane of the myotube (arrowheads) and are not prominent in lysosomes marked with LAMP-GFP. Scale bar: (A, B) 10 µm, (A′, B′) 5 µm. (TIF) [file pone.0114126.s007.tif]
